# Pancreatic ductal adenocarcinoma concomitant with pancreatic metastases of clear-cell renal cell carcinoma: a case report

**DOI:** 10.1186/s13256-021-02768-8

**Published:** 2021-05-31

**Authors:** Lena Haeberle, Melanie Busch, Julian Kirchner, Georg Fluegen, Gerald Antoch, Wolfram Trudo Knoefel, Irene Esposito

**Affiliations:** 1 Institute of Pathology, Heinrich Heine University and University Hospital Duesseldorf, Moorenstr. 5, 40225 Duesseldorf, Germany; 2 Department of Diagnostic and Interventional Radiology, Heinrich Heine University and University Hospital Duesseldorf, Duesseldorf, Germany; 3 Department of General, Visceral and Pediatric Surgery, Heinrich Heine University and University Hospital Duesseldorf, Duesseldorf, Germany

**Keywords:** Pancreatic cancer, Clear-cell renal cell carcinoma, Pancreatic metastases, Synchronous, Metachronous, Case report

## Abstract

**Background:**

Metastatic spread to the pancreas is a rare event. Renal cell carcinoma represents one possible site of origin of pancreatic metastases. Renal cell carcinoma often metastasizes late and exclusively to the pancreas, suggesting a special role of renal cell carcinoma among primaries metastasizing to the pancreas. Even rarer, renal cell carcinoma may occur simultaneously with pancreatic ductal adenocarcinoma.

**Case presentation:**

We present the case of a 78-year-old male Caucasian patient with a history of clear-cell renal cell carcinoma treated with oncological left nephrectomy 20 years before. The patient was diagnosed with pancreatic ductal adenocarcinoma by fine-needle aspiration cytology. At our institution, he received neoadjuvant therapy with folic acid, fluorouracil, irinotecan, oxaliplatin for borderline-resectable pancreatic ductal adenocarcinoma, and subsequently underwent total pancreatectomy. Upon resection, pancreatic ductal adenocarcinoma as well as two metachronous metastases of clear-cell renal cell carcinoma occurring simultaneously and cospatially with pancreatic ductal adenocarcinoma were diagnosed in the pancreatic body.

**Conclusions:**

Renal cell carcinoma metastases of the pancreas are rare and often occur decades after the initial diagnosis of renal cell carcinoma. The combination of renal cell carcinoma metastases and pancreatic ductal adenocarcinoma is even rarer. However, the possibility should be considered by clinicians, radiologists, and pathologists. The special role of renal cell carcinoma as a site of origin of pancreatic metastasis should be further elucidated.

## Background

Pancreatic ductal adenocarcinoma (PDAC) is the most frequent form of pancreatic neoplasm, accounting for approximately 85% of pancreatic tumors, and is most commonly localized in the pancreatic head [1]. Neoadjuvant therapy is emerging in PDAC treatment, especially in the context of borderline-resectable PDAC [2].

Pancreatic metastases are generally uncommon, with the most frequent sites of origin being lung cancer and gastrointestinal (GI) cancers [3]. More rarely, renal cell carcinoma (RCC) can spread to the pancreas, accounting for approximately 5% of all pancreatic metastases [3].

Here, we report the rare case of concomitant neoadjuvant-treated PDAC and two clear-cell RCC (ccRCC) metastases in the pancreas of a 78-year-old male patient and present a brief review of literature.

## Case presentation

A 78-year-old male Caucasian patient presented at our hospital with PDAC of the pancreatic body that had been diagnosed cytologically at a different institution. Due to newly occurring jaundice, he had received stenting of the distal bile duct and pancreatic fine needle aspiration (FNA) cytology. The patient had a history of oncological left nephrectomy for ccRCC 20 years prior. Relevant secondary diagnoses included permanent atrial fibrillation, hypertension, coronary artery disease, and chronic kidney failure. When the patient initially presented at our institution, he reported unintentional weight loss of 3–4 kg and a lack of appetite. Physical examination showed no remarkable findings.

**Fig. 1:**
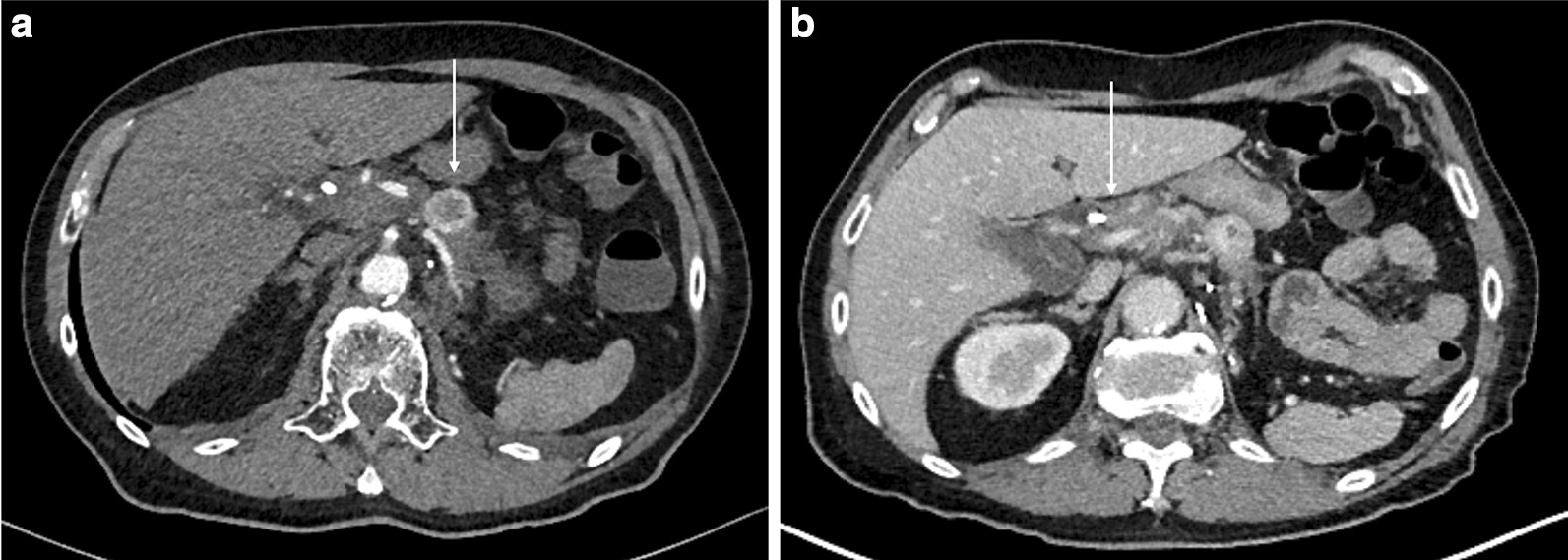
Computed tomography scans of the pancreas. **a** Hypervascular lesion in the body/tail of the pancreas (arrow), highly suspicious for a renal cell cancer metastasis or neuroendocrine tumor. **b** Hypodense, more proximally located lesion in the pancreas (arrow), suspicious for adenocarcinoma

During staging, abdominal sonography, computed tomography (CT), and chest x-ray demonstrated no distant metastases of PDAC. Multiple space-occupying masses in the pancreas were found on CT scans (Fig. 1). Due to radiologically suspected infiltration of the portal vein and possible abutment of the celiac trunk, the PDAC was classified as borderline resectable.

The patient received neoadjuvant chemotherapy with folic acid, fluorouracil, irinotecan, oxaliplatin (FOLFIRINOX) regimen because of good Eastern Cooperative Oncology Group (ECOG) performance status and borderline resectability of the PDAC. However, after the second FOLFIRINOX cycle, he developed a non-ST-segment elevating myocardial infarction (NSTEMI). Consequently, treating physicians decided to stop chemotherapy and proceed to surgical resection.

Subsequent positron emission tomography-computed tomography (PET-CT) scans showed the already known space-occupying masses in the body and tail of the pancreas. An additional, morphologically distinct area of high metabolic activity was seen in the pancreatic head.

The patient underwent total pancreatectomy with splenectomy and segmental portal/superior mesenteric vein resection and reconstruction, hemigastrectomy, and cholecystectomy.

**Fig. 2:**
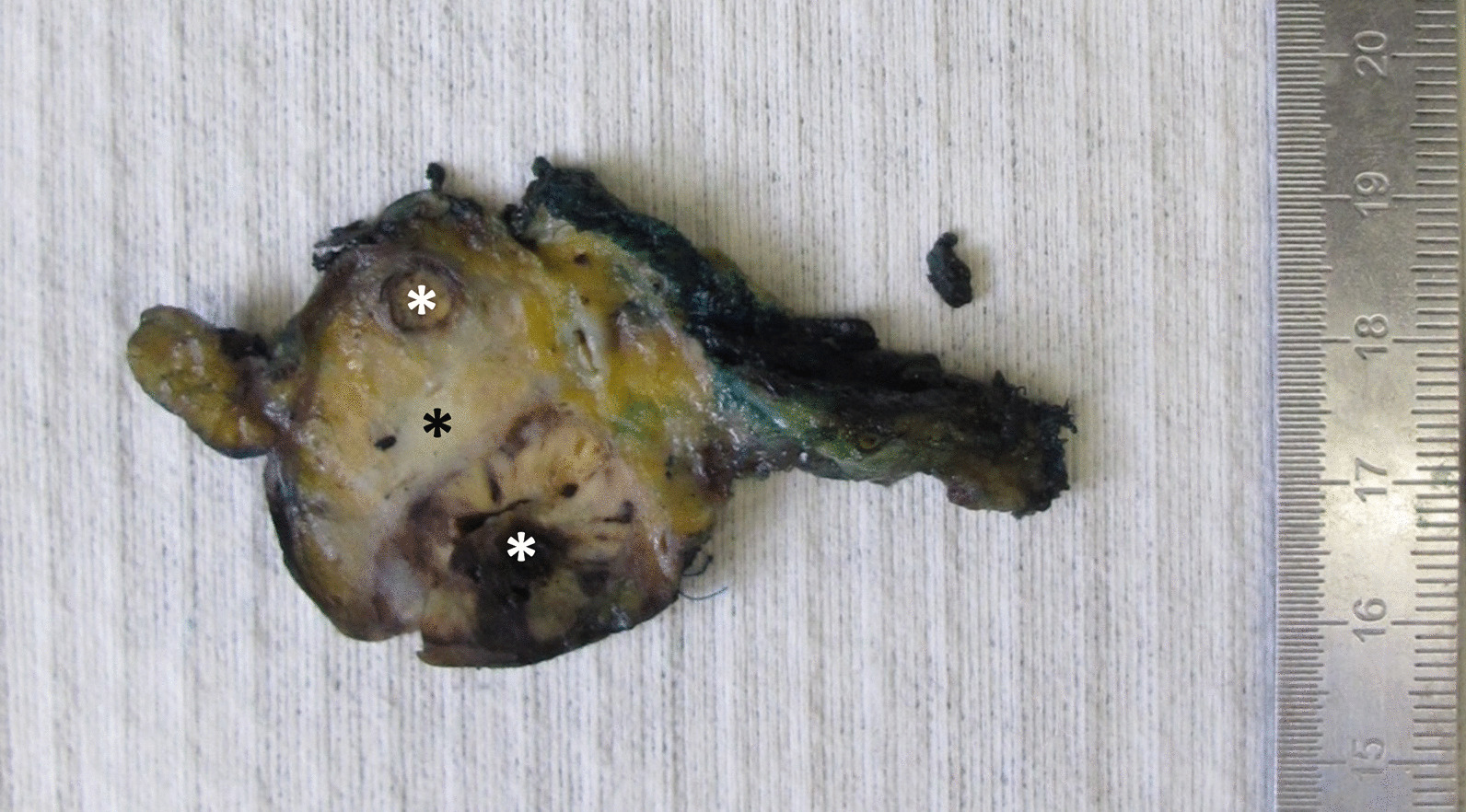
Macroscopic section of the pancreatic body. The two well-defined RCC metastases (white asterisks) as well as the ill-defined white-yellowish PDAC in between (black asterisk) can be seen. Axial slicing technique was used for grossing of the pancreatic head (not shown), while the pancreatic body and tail were sectioned parallel to the pancreatic neck margin

Upon pathological examination of the resection specimen, two well-defined tumors (1.8 cm in diameter and 0.4 cm in diameter, respectively) with inhomogeneous yellow and brown cut surface were found in the pancreatic body, encompassed by another ill-defined solid white-yellowish tumor with a diameter of 2.8 cm, extending to the pancreatic tail (Fig. 2).

**Fig. 3:**
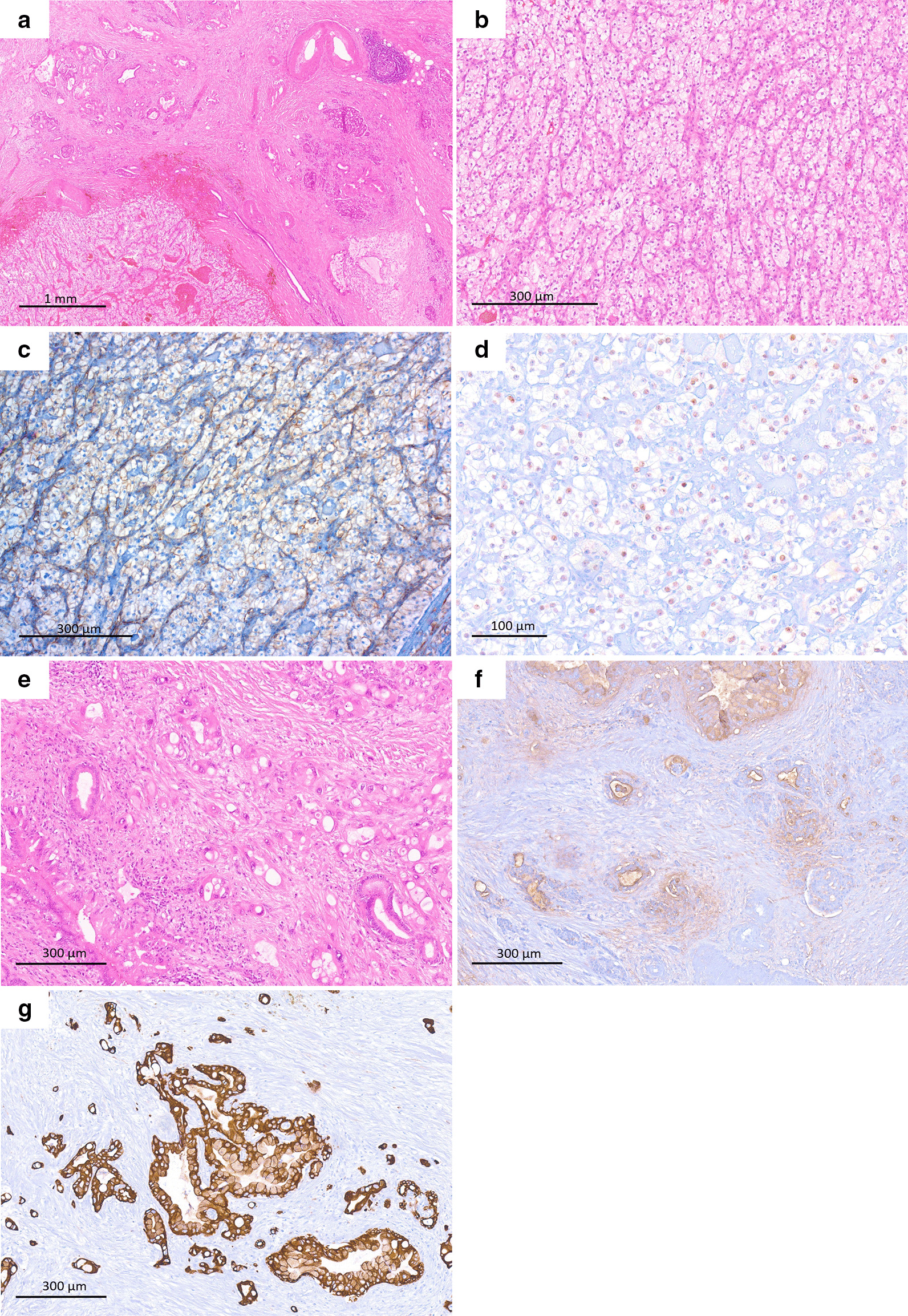
Histomorphology and immunohistochemistry of the pancreatic body/tail lesions.** a** Microscopic overview of ccRCC metastasis (bottom left) and PDAC (upper right) in immediate juxtaposition to one another (hematoxylin and eosin [H&E], 20×). **b** Higher magnification of ccRCC metastasis displaying its typical histomorphology with a solid growth of tumor cells with clear cytoplasm (H&E, 80×). **c** Immunohistochemistry for vimentin showing membranous positivity, confirming ccRCC metastasis (100×). **d** ccRCC metastasis also verified by nuclear positivity for Pax-8 in immunohistochemistry (200×). **e** Higher magnification of PDAC, containing irregular tubuli (bottom left), but also solid areas with vacuolized eosinophilic cytoplasm and highly pleomorphic nuclei, accounting for regressive changes after neoadjuvant therapy (upper right) (H&E, 80×). **f**, **g** PDAC tumor cell complexes showing characteristic positivity for Ca19-9 (**f**) and CK7 (**g**) on immunohistochemistry (80×)

Upon microscopic examination, tumor cell formations with different histomorphology were found in the pancreatic body/tail (Fig. 3a). The two well-defined tumors shared an identical histomorphology displaying nodular growth of uniform tumor cells with transparent cytoplasm and small nuclei (Fig. 3b). Upon immunohistochemistry, the tumor cells stained positive for vimentin and Pax-8 (Fig. 3c, d), whereas they were negative for CK7 and Ca19-9, and were therefore diagnosed as ccRCC metastases. The larger, ill-defined tumor extending to the pancreatic tail showed a more heterogeneous histomorphology. While some areas displayed small- to medium-sized irregular tubular glands embedded in a desmoplastic stroma, other areas showed a cribriform to solid growth pattern and consisted of tumor cells with prominent eosinophilic cytoplasm, sometimes containing cytoplasmic vacuoles and highly pleomorphic enlarged nuclei (Fig. 3e). Upon immunohistochemistry, these tumor cells expressed Ca19-9 and CK7 (Fig. 3f, g), while staining negative for vimentin and Pax-8, thereby representing PDAC. Adenosquamous differentiation was excluded by p40 immunohistochemistry (not shown). Hypereosinophilic cytoplasm and cytoplasmic vacuoles were interpreted as regressive cytopathic changes following preoperative chemotherapy. The tumor regression was classified as grade 3 (poor response) according to the College of American Pathologists (CAP) tumor regression grading system [4]. Moreover, numerous venous and perineural infiltrations, six regional lymph node metastases, and peritoneal carcinomatosis were found. The final TNM stage of the PDAC was ypT2 ypN2 (6/45) ypM1 (PER) L0 V1 Pn1 R1.

The patient initially recovered steadily after surgery. However, approximately 2 weeks after surgery, he suffered from pulmonary aspiration and had to receive CPR and bronchoscopic suctioning. In a subsequent abdominal CT scan, free intraabdominal gas was detected, prompting surgery including atypical partial resection of the stomach due to ischemic perforation. Although surgery was successful, the patient developed septic shock with disseminated intravascular coagulation the following day. Despite further surgery, including subtotal colectomy due to ischemia, the patient passed away one day later as a result of refractory shock and multiple organ failure.

An overview of the clinical course is given in Table 1.

**Table 1:** Overview of the clinical course in the present case

Time point	Event
*T* = 0	ERCP with stenting of distal bile duct and EUS-FNA cytology of the pancreatic body due to painless jaundice (*ex domo*)
*T* = 5 days	Diagnosis of G2 PDAC of the pancreatic body in EUS-FNA cytology material (*ex domo*)
*T* = 5 weeks	Begin of FOLFIRINOX chemotherapy with neoadjuvant intent due to borderline resectability of PDAC and patient’s good general condition
*T* = 7 weeks	NSTEMI with subsequent coronary artery stenting and stop of FOLFIRINOX therapy
*T* = 8 weeks	Interdisciplinary decision for surgical therapy
*T* = 10 weeks	Total pancreatectomy with splenectomy and segmental portal/superior mesenteric vein resection and reconstruction, hemigastrectomy and cholecystectomy
*T* = 12 weeks	Worsening postoperative condition, including ischemic perforation of the stomach, colon ischemia, refractory shock, and multiple organ failure, leading to the patient’s death

*ERCP* endoscopic retrograde cholangiopancreatography, *EUS-FNA* endoscopic ultrasound-guided fine-needle aspiration, *FOLFIRINOX* folinic acid, fluorouracil, irinotecan, oxaliplatin, *NSTEMI* non-ST-segment elevating myocardial infarction, *PDAC* pancreatic ductal adenocarcinoma

## Discussion

Metastases to the pancreas are rare and are often not clinically apparent. In a study considering surgical specimens and autopsy data, most resected pancreatic tumors were primary pancreatic tumors (such as PDAC), while only 3.9% of pancreatic surgical specimens harbored tumors of extrapancreatic origin, involving the pancreas secondarily [3]. However, 43% of the pancreatic tumors found in autopsy cases were found to be secondary tumors, suggesting that secondary tumors in the pancreas are often clinically inapparent [3]. While in the autopsy series, lung cancer (42%) and GI cancers (25%) were the most frequent sites of origin for metastases in the pancreas, the surgical series contained mainly lymphomas (29%) and carcinomas of the stomach (18%) and kidney (16%) as sites of origin of pancreatic metastases [3]. A systematic literature review found that, among patients who received pancreatectomy for metastases, RCC metastases were the most frequent (63%) [5].

The median interval between primary RCC and metastasis to the pancreas is approximately 7 years, and pancreatic metastasis has been shown to occur as late as 20 years after the primary diagnosis of RCC [6]. The fact that pancreatic metastases of RCC often occur late, taken together with the fact that the pancreas is frequently the only organ to be affected by metachronous RCC metastases, has prompted authors to suggest a “seed and soil” theory in the context of RCC metastases to the pancreas. Accordingly, disseminated tumor cells spread through the vascular system and may only develop into metastases at locations where specific interactions between tumor cells and host tissue allow for maturation to metastases, for example, facilitated by the formation of a premetastatic niche, specific chemokine interactions between tumor cells and host tissue, and tumor-cell-favoring immunoediting in/of the host tissue [7]. However, the exact reasons for the “pancreatotropism” of RCC cells remains unknown. Patients resected for pancreas metastases from RCC seem to have a significantly more favorable prognosis compared with patients resected for pancreas metastases of other origin [5]. This is in line with findings indicating that ccRCC with pancreatic metastasis is characterized by high vascularization, homogeneous growth pattern, and low-grade nuclei in histopathology as well as a less aggressive molecular profile compared with ccRCC with nonpancreatic metastases [8]. This suggests that ccRCC with (exclusive) metastases to the pancreas represent a specific rather indolent subset of ccRCC. The above-described histomorphological aspects can also be found in the present case (Fig. 3).

In the present case, late metachronous RCC metastasis were found concomitant with PDAC. Patients with RCC generally seem to have a significantly higher risk of developing various other primary malignancies [9]. As chemotherapy and/or radiation are usually not part of the initial therapy of RCC, it can be hypothesized that these additional primary malignancies in RCC patients are not therapy induced, but rather linked to exogenous and/or genetic factors. For example, tobacco smoking is a risk factor shared by various cancers, including RCC and PDAC. Pathogenic variants of *VHL* (Von Hippel–Lindau tumor suppressor) are a major genetic parameter linked to ccRCC. Patients with VHL disease harbor an increased risk of developing ccRCC, among other tumors [10]. In addition, 46–82% of sporadic ccRCC contain pathogenic mutations of *VHL* [10]. Patients with VHL disease are also prone to develop serous cystic neoplasms (SCN) of the pancreas and pancreatic neuroendocrine neoplasms (pNEN); however, neither VHL disease and nor sporadic *VHL* variants are linked to PDAC [11].

Cases of synchronous or metachronous RCC and PDAC in the pancreas have rarely been reported in the literature. In 2012, a retrospective study of 1178 patients with pancreatic cancer found 16 cases of PDAC or intraductal papillary mucinous neoplasm (IPMN) in the pancreas with synchronous or metachronous RCC. Among 12 PDAC patients, 9 patients with metachronous RCC, but only 3 patients with synchronous RCC, were identified [12]. In Table 2, an overview is given of the available cases in the English-speaking literature, with detailed information on simultaneously occurring PDAC and RCC.

**Table 2:** Overview of literature on simultaneously occurring pancreatic ductal adenocarcinoma (PDAC) and renal cell carcinoma (RCC)

Age	Sex	Subtype and localization of RCC	Procedure for RCC	Localization of PDAC	Procedure for PDAC	Follow-up	Source
78	M	ccRCC, two pancreas body metastases	Total pancreatectomy	Pancreatic body/tail	Total pancreatectomy	Perioperative death*	Present case
70	M	ccRCC, kidney (exact site unknown)	Partial nephrectomy	Proximal pancreas	ppWhipple	35 months, alive	[12]
70	M	ccRCC, kidney (exact site unknown)	Partial nephrectomy	Proximal pancreas	ppWhipple	5.25 months, dead	[12]
71	M	Papillary RCC, kidney (exact site unknown)	Partial nephrectomy	Proximal pancreas	ppWhipple	3.25 months, dead	[12]
67	M	ccRCC, left kidney	Left nephrectomy	Pancreas head	ppWhipple	15 months, dead	[13]
62	M	Papillary RCC, right kidney	Radical right nephrectomy	Pancreas body	Unknown; not resected	Unknown	[14]
70	F	ccRCC, right kidney	Chemotherapy	Pancreas body	Chemotherapy	13 months, dead	[15]

*PDAC* pancreatic ductal adenocarcinoma, *(cc)RCC* (clear-cell) renal cell carcinoma, *F* female, *M* male, *pp* pylorus-preserving

*Death 15 days after surgery

The strength of our case report resides in the detailed radiological and pathological evaluation of the simultaneously and cospatially occurring PDAC and pancreatic ccRCC metastases. However, its limitation is the short follow-up period due to the patient’s postoperative death. Of course, reasons for and against surgery must be carefully weighed in a case with extensive involvement of the pancreas by multiple masses in a patient with relevant comorbidities and reduced general health condition. Decision for surgery was made in the present case in an interdisciplinary tumor board despite the patient’s advanced age and recent myocardial infarction during FOLFIRINOX therapy, as PDAC was classified as borderline resectable, he was in stable general condition, and he expressed an explicit wish to undergo surgery.

In the present case, the primary RCC occurred antecedent to the primary PDAC; however, RCC metastases and PDAC occurred simultaneously and in the same location. It is speculative whether in this case the presence of PDAC might have played a role in the development of pancreatic RCC metastasis, for example, by creating a favorable local immune environment, or vice versa.

Generally, the case presented here illustrates that, although preoperative diagnostic tools have reached a high standard of quality, the validity of a preoperative diagnosis can still be limited, underlining the importance of pathological examination in the diagnostic process.

## Conclusions

Although RCC metastases in the pancreas are rare, and their combination with PDAC even more so, clinicians, radiologists, and pathologists should be aware of this possibility. Interpretation of imaging in such cases may be highly challenging, especially after preoperative treatment. Hence, close interdisciplinary collaboration is essential in the diagnostic process.

## Data Availability

All data generated or analyzed during this study are included in this published article.

## Abbreviations

Ca 19-9: Carbohydrate antigen 19-9
(cc)RCC: (Clear-cell) renal cell carcinoma
CK7: Cytokeratin 7
CPR: Cardiopulmonary resuscitation
(PET-)CT: (Positron emission tomography-)computed tomography
ECOG: Eastern Cooperative Oncology Group
ERCP: Endoscopic retrograde cholangiopancreatography
(EUS-)FNA: (Endoscopic ultrasound-guided) fine-needle aspiration
FOLFIRINOX: Folic acid, fluorouracil, irinotecan, oxaliplatin
GI: Gastrointestinal
IPMN: Intraductal papillary-mucinous neoplasm
NSTEMI: Non-ST-segment elevating myocardial infarction
Pax-8: Paired box protein 8
pNEN: Pancreatic neuroendocrine neoplasm
PDAC: Pancreatic ductal adenocarcinoma
SCN: Serous cystic neoplasm
VHL: Von Hippel–Lindau
